# Uptake of community blood pressure monitoring and associated factors among people living with HIV and hypertension accessing care at selected HIV clinics in Urban and Peri-urban Uganda

**DOI:** 10.21203/rs.3.rs-8426924/v1

**Published:** 2026-01-29

**Authors:** John Baptist Kiggundu, Rawlance Ndejjo, David Guwatudde, Fred C. Semitala

**Affiliations:** 1 Infectious Diseases Research Collaboration, Kampala, Uganda; 2 Department of Disease Control and Environmental Health, School of Public health, Makerere University, Kampala, Uganda; 3 Department of Epidemiology and Biostatistics, School of Public health, Makerere University, Kampala, Uganda; 4 Department of Medicine, Makerere University, Kampala, Uganda; 5 Makerere University Joint AIDS Program, Kampala, Uganda

## Abstract

**Introduction:**

The World Health Organization recommends Community-based blood pressure monitoring (BPM) for diagnosis and management of hypertension. However, there is limited data on the uptake and factors associated with community BPM among people living with HIV (PLHIV) and hypertension in many low-income settings including Uganda. This study aimed to determine the uptake of, and factors associated with community BPM among PLHIV and hypertension receiving care at selected HIV clinics.

**Methods:**

A cross-sectional study was conducted between May and July 2024 at three HIV clinics participating in an implementation trial [(NCT05609513), registration date, November 8, 2022) in the Kampala and Wakiso districts. A total of 408 participants, proportionally distributed across the clinics, were randomly selected and surveyed using a pretested questionnaire. Descriptive statistics were performed, reporting means or medians with standard deviations (SD)/interquartile ranges (IQR) and frequencies with proportions for numerical and categorical variables, respectively. Factors associated with community BPM uptake were assessed using bi-variable and multivariable modified Poisson regression. Data analysis was performed using STATA 14.1

**Results:**

Of the 408 participants, 67.7% were female, mean age of 50.1 years (SD=10.7). All participants had been on HIV and hypertension medications for a median duration of 5 years (IQR: 3.5–7.2) and 1 year (IQR 1–1), respectively. Community BPM uptake was 27% (95% CI: 23.3–32.0). Among those monitoring their BP in the community, 68.6% did so at private clinics, 70% at pharmacies, 15.2% at public health facilities, and 5.4% self-monitored their BP. Multivariable analysis indicated that the uptake of community BPM was associated with receiving three antihypertensive medications (adjusted prevalence ratio [aPR]: 2.2, 95% CI: 1.1–4.4), receiving advice from healthcare providers (aPR: 8.7, 95% CI: 3.7–20.5), receiving feedback on BP monitoring (aPR: 33.3, 95% CI: 4.1–268.6), and owning a BP device (aPR: 2.2, 95% CI: 1.3–3.5).

**Conclusion:**

Community BPM uptake among PLHIV and hypertension was low. However, individuals receiving healthcare provider recommendations and feedback, owning BP devices, or on three antihypertensive medications were more likely to engage in community BPM. To improve uptake and ensure continuity of care, targeted interventions such as healthcare provider counseling, feedback mechanisms, and facilitating access to BP devices are recommended.

## INTRODUCTION

HIV infection and hypertension remain major public health concerns in Uganda and across the globe. Globally, 40 million people are living with HIV, of whom more than half reside in Eastern and Southern Africa. An approximately 20-30% of the estimated 1.4 million adult persons living with HIV (PLHIV) in Uganda are also living with hypertension (1-4). While vertical HIV programing has significantly improved HIV care metrics over the past four decades—as evidenced by high rates of HIV diagnosis (81%), treatment initiation (96%) and viral load suppression (92%), hypertension care metrics remain alarmingly poor(5, 6). Although hypertension is a major modifiable risk factor for cardiovascular disease (CVD), it remains frequently underdiagnosed and undertreated among PLHIV, with suboptimal blood pressure (BP) control even among those receiving treatment. (7). Recent estimates from Uganda’s primary healthcare facilities suggest that only 27% of PLHIV were screened for hypertension, of whom 46% were diagnosed and only 24% had achieved adequate BP control(6, 7).

Given the high prevalence of hypertension and the suboptimal performance across the hypertension care cascade among PLHIV, the Uganda Ministry of Health (MoH), in alignment with World Health Organization (WHO) guidelines, recommends the integration of HIV and hypertension services(8, 9). However, the distinct clinical demands of hypertension care may pose challenges to effective service integration. For example, most adult PLHIV require viral load monitoring only once a year, whereas persons living with hypertension, regardless of BP control status, require more frequent BP assessments. Community-based BP monitoring, combined with remote transmission of readings to healthcare providers is recommended for regular BP surveillance without necessitating frequent in-person visits to HIV clinics.

BP monitoring is a vital component of hypertension self-care practice. BP monitoring facilitates titration of anti-hypertensive medications, which leads to improved BP control. Regular monitoring also contributes to better BP control among individuals living with hypertension (10-12). In addition, community BP monitoring is essential for diagnosis of masked hypertension, white coat effect and reduces health care provider’s reliance on one-time clinic BP measurements which are often influenced by environment and psychological factors, might not reflect BP on day-to-day BP levels (13). The prevalence of self BP monitoring varies significantly by geographic region, ranging from 40% to 90% in high-income countries (HICs) (14, 15). In contrast, self BP monitoring among individuals with hypertension in sub-Saharan Africa—where approximately one-third of the global hypertensive population resides—remains notably low. Recent studies conducted in specialized hypertension clinics across Africa report that only 8.9% to 36% of patients monitor their BP at home (16). Although home, self- and ambulatory BP monitoring are the most widely recommended approaches to out of clinic BP monitoring, their implementation in low resourced countries, such as Uganda remains low due to limited access to personal BP monitoring devices, making these approaches less feasible in practice (17, 18) .

As part of the ongoing Strengthening Blood **P**ressure Care and Treatment Cascade for **U**gandans Living with HIV—Imp**LE**mentation Strategies to **SA**ve Lives (PULESA-Uganda) study(19), we promoted out-of-clinic BP monitoring to ensure that patients continue to have their BP assessed even when they are away from the HIV clinic, thereby supporting continuity of care. Healthcare providers encouraged and educated patients on the importance of community-based BP monitoring through clinic health education sessions and one-on-one patient-provider interactions. Healthcare providers were also trained on hypertension care and treatment and patients received anti-hypertensive medication from the clinics at no-cost. In this study, we defined community BP monitoring as BP measurements taken and recorded from outside the primary HIV-hypertension clinic setting (i.e. at home, a nearby public or private medical facility other than the primary HIV clinic, a private pharmacy, or through the use of shared community BP machines). To facilitate this practice, healthcare providers only distributed BP documentation cards or journals and encouraged patients to record their BP readings obtained in community settings. No BP devices were given to patients. Patients were further advised to share these readings with healthcare providers either through the clinic’s telephone lines or during subsequent clinic visits. This cross-sectional study sought to evaluate the prevalence of BP monitoring, examine approaches to community-based BP monitoring, and identify factors influencing its adoption among persons with HIV and hypertension using the Anderson healthcare utilization model(20).

## METHODS

### Study design

We conducted a cross-sectional study at three selected HIV clinics offering integrated HIV-hypertension care in Kampala and Wakiso districts, Uganda. This study was nested in an ongoing PULESA Uganda trial [(NCT05609513), registration date, November 8, 2022], which aims to evaluate strategies for integrating hypertension care into HIV services in Kampala and Wakiso districts in Uganda (19).

### Study setting

We purposively selected three clinics out of the eight clinics implementing the enhanced package for delivery of integrated HIV-hypertension care in the PULESA Uganda trial. Kawala HCIV HIV clinic is located at a public health center IV in Kampala and provides care to 8,000 PLHIV, of whom 651 had comorbid hypertension. Butabika Hospital HIV clinic is situated at a national referral hospital (mental) located in Kampala, offers comprehensive HIV care to about 1120 PLHIV and 156 PLHIV with hypertension. Nakawuka HCIII is peri-urban clinic located in Wakiso district serves about 839 with 139 having comorbid hypertension. The study was conducted between May 2024 to September 2024. At these clinics, HIV and hypertension services have been integrated such that patients with comorbid conditions receive health lifestyle counselling, clinic BP monitoring, HIV and antihypertensive medications prescriptions and refills provided by the same healthcare provider in the same setting as HIV services. Both HIV and hypertension services are provided at no cost to the recipients of care.

### Participant selection

Participants receiving HIV and hypertension care were approached by a research assistant either before or after their clinical encounter with a healthcare provider to asses eligibility to participate in the study. PLHIV enrolled in HIV care at one of the study clinics, aged 18 years or older, and have a confirmed diagnosis of hypertension made prior to enrollment were considered eligible for the study. Hypertension diagnosis was defined as BP ≥140/90 mmHg on two or more separate occasions or current use of anti-hypertensive medication specifically for hypertension as per the Uganda national guidelines. Participants were excluded if they had any of the following criteria; had advanced cognitive impairment or another condition that significantly impaired their ability to complete study assessments; were taking anti-hypertensive medication solely for an indication other than hypertension.

### Sample size and sampling

We estimated the sample size using the sample size for formula for cross-sectional studies, assuming a prevalence (P) of self-blood pressure monitoring of 36%, 95%, confidence level of 5% (z= 1.96), and maximum error (δ) of 5%. Given that this was a clustered cross-sectional study, the sample size was adjusted for design effect. After adjusting for clustering, a sample of size of 406 participants was obtained and proportionately distributed across the three study clinics.

At each clinic, participants receiving integrated HIV and hypertension care were consecutively sampled and approached by a research assistant, either before or after their visit with the clinician. Enrollment began at all three clinics around the same time and continued until the clinic target sample size was reached. The sample size for each clinic was calculated using the proportionate sampling, proportions calculated based on the number of patients registered at each of the clinics. This approach ensured that participants were recruited from each clinic in equal proportions. Table 1.

### Study variables and data collection

Four trained research assistants with background training in nursing and social sciences administered a pretested questionnaire. The questionnaire was translated in Luganda, a commonly spoken language.

The dependent variable, “community BP monitoring”, was defined as documented community BP measurement within the past 30 days from the date of the survey; “1-Yes” – was recorded if the participant had a documented BP measurement done outside the primary clinic setting whereas “0-No”- was recorded if the participant had no recorded BP measurement outside the primary clinic setting within 30 days before the survey date.

Independent variables were categorized into; Predisposing factors, Enabling / disabling factors, and Perceived or Evaluated need using the Andersen healthcare utilization model.(20) ; as described below:

- **Predisposing factors: Included** Age (number), Sex (male or female). Education level (No education, primary level, secondary), Occupation (not employed, formal employment), monthly income status (number), healthcare provider recommendation (Yes/No), advise to monitor BP in the community (Yes/No), healthcare provider provides feedback on BP monitoring (Yes/no), smoking status (Yes/No), alcohol consumption (Yes/No). All the above variable was collected by asking the participants. Duration of hypertension or HIV (number) was estimated using by establishing the date of diagnosis in the participants medical records.
- **Enabling / disabling factors:** Knowledge about the importance of self-monitoring (Low/ good knowledge- details about the assessment are provided in analysis section), Peer and treatment support (yes/no), Knowledge about hypertension (good/low knowledge),attitude about BP monitoring (good or poor attitude-details about the assessment are provided in analysis section) proximity to the health facility (distance to the clinic -number), ownership of BP monitoring device (Yes/No), knowledge about hypertension treatment goals (Yes/No), access to journal(book/card) for documenting BP measurements ( yes/no)
- **Perceived or Evaluated need:** Frequency of clinic visits, Availability of opportunity to review and document BP in patients records, comorbidity [diabetes (Yes/No), kidney disease (Yes/No)], hypertension severity (controlled /uncontrolled), hypertension medications (number). These data were obtained by reviewing the participants medical records.

To assess participants' attitudes towards BP monitoring, we used a Likert scale with five possible responses: strongly agree (5), agree (4), neutral (3), disagree (2), and strongly disagree (1). Participants were presented with four sentences and asked to respond using this scale.

1. I feel confident I can have access to community BP monitors without trouble
2. I believe community BP monitoring will make a difference in controlling my BP
3. I believe BP readings from the community are not as accurate as those from the clinic here.
4. I would recommend others to use community monitoring of BP

To assess participants' knowledge about BP monitoring, we asked four questions with the following responses “Yes”, “Don’t know” and “Not sure”. “Yes” was coded as 1 and Do not know and Not sure responses were coded as zero. A sum of the aggregates was obtained and categorized into low knowledge and high knowledge about BP monitoring.

1. Monitoring my BP regularly will encourage me to take my medicines regularly.
2. Monitoring my BP will enable me to achieve the desired BP targets
3. BP readings from away from the clinic will be compared with clinic readings by the provide to get an accurate picture of my actual BP

The survey data were collected and managed using the Research Electronic Data Capture (REDCap), with in-built commands to minimize data entry errors. REDCap database is hosted at the Infectious Diseases Research Collaboration (21, 22).

### Data management and analysis

Participation in community BP monitoring was summarized as the proportions of participants who had community BP measurement with a 95% confidence interval (CI). Normally distributed age and systolic and diastolic BPs were summarized using mean and standard deviation (SD) while non-normally distributed continuous variables (duration of hypertension diagnosis, time spent on anti-hypertensive and ART medications, and distance were summarized as medians and IQR. For categorical variables such as biological sex, level of education, presence of comorbidities, prior history of hypertension, viral load status, and level of education were summarized using frequencies, and percentages.

We assessed participants’ attitudes toward blood pressure (BP) monitoring by calculating a total score, obtained by summing responses to each item. Scores ranging from 4 to 15 indicated a poor attitude, while scores from 16 to 20 reflected a good attitude.

To evaluate participants’ knowledge of BP monitoring, we similarly computed a total score by summing responses to each item. Scores between 0 and 2 were categorized as low knowledge, whereas scores between 3 and 4 were considered high knowledge. No previously validated questionnaires were available for these assessments.

### Multivariable analysis

The primary outcome, community BPM was dichotomized and coded “1” for participants who had monitored their BP in the community within the past 30 days from the day of the interview and “0” for participants who had not.

Multi-variable modified Poisson regression was performed to assess factors associated with the community BP monitoring, as it provides prevalence ratios, which are more accurate estimates than odds ratios from logistic regression in studies with common outcomes. (23, 24). Independent variables were assessed for multicollinearity using correlation coefficients to avoid model overfitting. Variables with p-values ≤ 0.2 were included in a correlation matrix, and where pairs had coefficients ≥ 0.4, one was excluded. Income level and feedback from healthcare providers were retained over clinician recommendations due to collinearity. Non-collinear variables were included in the multivariable analysis with the outcome variable.

A multivariable model was run including all the independent variables and their interaction terms and was taken as the “full model”. Another multivariable model was run with only the basic variables and was taken as the “reduced model”. A likelihood-ratio (chunk) test comparing the full model to the reduced model was used to check for interaction and statistically significant interaction was present. Interaction terms that were not statistically significant were removed from the full model, one at a time, until only the statistically significant interaction terms remained. The model was then adjusted for confounding by removing the basic variables whose associations to community BP monitoring were not statistically significant, one at a time. Finally, the prevalence ratios (PR) obtained after this process were reported as adjusted prevalence ratios along with their 95% confidence intervals and *P*-values. Data were analysed in Stata version 14.1 (STATA CORP, Texas, USA).

### Ethical Considerations

This research was conducted in accordance with the Declaration of Helsinki.Participants provided written informed consent before participating in the study. Enrolment into the study was voluntary and participants had the right to withdraw at any time from the study. Ethical approval to conduct the study was obtained from the Makerere University School of Public Health Research Ethics Committee (MakSPH-REC 354). Additionally, permission was sought from the PULESA Uganda trial leadership to nest this sub-study within the main trial.

### Supplementary material

We adhered to the Strengthening the Reporting of Observational Studies in Epidemiology (STROBE) guidelines for reporting cross-sectional studies. **Supplement material 1.**

## RESULTS

### Characteristics of participants

#### Eligibility screening, enrollment

**Figure 1:**
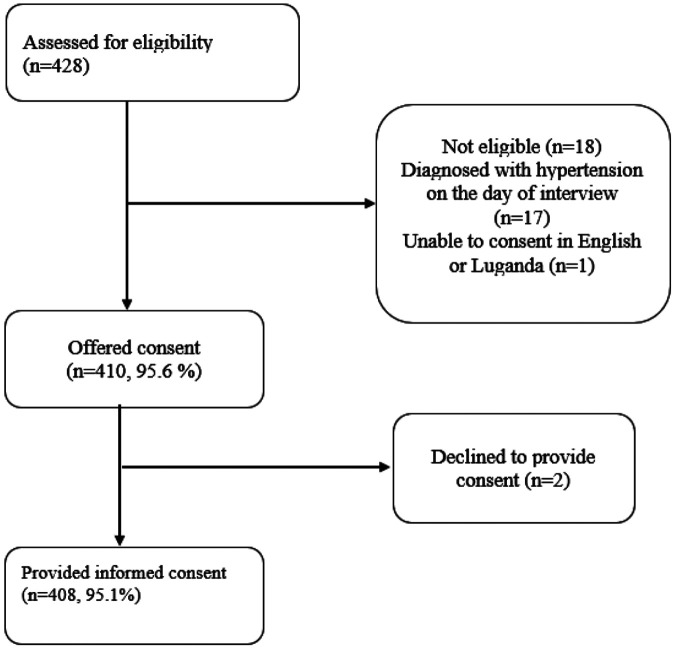
Summary of study participant eligibility screening and enrollment

From 15 May 2024 to 29 September 2024, a total of 429 PLHIV and hypertension were assessed for eligibility to participate in this survey. Of these, 410 (95.6%) met the eligibility criteria, and 408 (95.1%) provided written informed consent and were surveyed. Figure 1.

### Participants’ socio-demographic characteristics

The majority of the participants were receiving care from Kawaala HCIV 282/408 (69.3%). The overall mean age was 50.1 years (SD=10.7) with the participants from Nakawuka having a significantly younger, mean age of 48 years (SD=13.2). Overall, almost half of the participants were 51 years and above. In Kawaala, Butabika and Nakawuka, 47.2% (133/282), 59.7% (47/67) and 39% (23/59) were aged 51 years and above. Among respondents, more than two-thirds, 276/408 (67.7%) were females, and most of them had attained secondary 134/408 (33.8%) or post-secondary education 59/408 (14.5%). Almost half of the participants, 188/408 (46.1%) were self-employed and their median monthly income was Uganda Shillings (UGX) 300,000 (IQR 122,000-600,000). Participants from Butabika and Nakawuka had significantly lower monthly income. Table 2.

### Participants’ clinical characteristics

All participants were on antiretroviral therapy with 92.2% (376/408) on TDF/3TC/DTG. The median duration on ART was five years (IQR: 3.5-7.2). Most participants had achieved viral load suppression 354/408 (86.8%) and only 22 (5.4%) having low-level viremia (200 −1000copies/ml). More than half of the participants were either overweight 125 (30.6%) or obese 108 (26.5%). The majority of the participants had been diagnosed with hypertension for about 1 year 231 (56.6%) and all participants were on antihypertensive medicines. Fifty-three per cent (217/408) were receiving two classes of anti-hypertensive medications and 137 (33.6%) had a systolic and diastolic BP below 140 and 90 mmHg at the previous clinic visit. Only one in ten (n=41) had another comorbid condition other than HIV and hypertension, with diabetes being the most common 26 (6.4%). Table 3.

### Healthcare system and behavioral related factors

About three-quarters of the participants, 75.1% (305/408), reported being advised by a healthcare provider to monitor their BP in the community. Almost all participants 94.4% (356/408), believed it was important for people living with hypertension to regularly monitor high BP while in the community. However, only 62.3% (254/408) reported having a journal (book or card) where they could document their community BP measurements, and 54.4% (222/408) mentioned that they were asked by their healthcare providers about these measurements on a subsequent clinic visit. Only 3.9% (16/406) owned a personal BP device. Table 4.

### Uptake of community BP monitoring

About a quarter, 112/408 (27.5%), 95% CI: 23.3-32% of the surveyed participants had monitored their BP in the community within the last 30 days prior to the date of interview.

The median frequency of community BP monitoring was 3 times (IQR: 2-4) a month. Most participants monitored their BP from a nearby private clinic, 77/112 (68.6%) while 70/112 (63.1%) paid to have their BP measured. Table 4. The proportion of participants who engaged in community BP monitoring was slightly higher than the overall average at Kawala (29.1%, 82/282) and Nakawuka (30.5%, 18/59), while fewer participants monitored their BP in the community at Butabika (17.9%, 12/67). However, these differences in community BP monitoring uptake across the clinics were not statistically significant.

### Factors associated with community BP monitoring analysis

At multivariable analysis, participants receiving three or more classes of antihypertensive medications were 2.2 times more likely to monitor their BP in the community compared to those taking only one class of antihypertensive medication (aPR: 2.2, 95% CI: 1.1-4.4, p=0.03). Although participants taking two classes of antihypertensive medications also demonstrated higher likelihood of community BP monitoring, this association did not reach statistical significance. The prevalence of community BP monitoring was significantly higher among participants who received advice from healthcare provider to monitor their BP in the community (aPR: 8.7, 95% CI: 3.7-20.5, p<0.0001) and those who received feedback on their BP monitoring practices (aPR: 33.3, 95% CI: 4.1-268.6, p<0.0001). BP ownership was also positively associated with community BP monitoring. Specifically, participants who owned personal BP devices were 2.2 times more likely to monitor their BP in the community compared to those without a personal BP device (aPR: 2.2, 95% CI: 1.3-3.5, p=0.002). Conversely, participants who were aware of at least one hypertension-mediated organ damage (HMOD) had a 30% lower likelihood of engaging in community BP monitoring (aPR: 0.7, 95% CI: 0.5–1.0, p = 0.07). However, this association did not reach statistical significance. Table 6.

## DISCUSSION

This cross-sectional study aimed to determine the uptake of, and factors associated with, community BP monitoring among PLHIV and hypertension receiving HIV-hypertension care at three PHC HIV clinics. One in every four of the participants reported engaging in community BP monitoring, with the majority preferring to monitor their BP at private community clinics, while fewer participants reported self-blood pressure monitoring. Factors associated with community BP monitoring were the use of three or more antihypertensive medications, having received advice from a healthcare provider to monitor BP in the community, receiving feedback from healthcare providers regarding community BP measurements, and owning a personal BP device.

Overall, about a quarter of the participants monitored their BP in the community. This proportion is consistent with the 25%-36% range reported in studies from Ghana, Cote d’Ivoire and China (25-27). This study reports a higher proportion of participants monitoring their BP in the community compared to the 7.7% and 8.9% reported in studies conducted in two Ethiopian states (16, 28). This difference can be attributed to the fact that our study combined the proportions of those who monitored their BP at community private clinics, pharmacies, or at home, unlike the aforementioned studies which focused on self-BP monitoring. Additionally, this study was conducted in HIV clinics that provided integrated HIV and hypertension care, unlike the previously mentioned studies, which were primarily conducted in hypertension clinics. The findings indicate that BP monitoring can be achieved using various community-based approaches, even in areas with limited access to personal BP devices. In our study, over two-thirds of participants monitored their BP at private clinics, while only 7% used home BP monitoring. Given the low availability of personal BP devices, promoting alternative, more accessible, and cost-effective BP monitoring methods within communities is essential.

Participants who were advised by their healthcare providers to monitor their BP within the community were eight times more likely to do so. Table 6. Similarly, Wake et al. reported that patients who received a healthcare professional’s recommendation to monitor their blood pressure were six times more likely to do so(28). Likewise, Ostchega found an eightfold increase in blood pressure monitoring among patients who received such recommendations(29). This increased likelihood is probably due to patients’ trust in their healthcare providers, leading them to perceive providers’ recommendation as important and eventually feel empowered and the need to carry on with the behavior. Participants who received feedback on their BP monitoring practices were significantly more likely to continue monitoring their BP within the community than those who did not receive such feedback. Although this association is strong, it needs to be interpreted in light of wider confidence interval which reflects variability in the data. Regardless, receiving feedback about practices or behaviors likely reinforces the importance of BP monitoring and may provide patients with valuable information regarding their BP measurements and whether they have achieved the desired goals or not(30). Feedback could also help patients understand the impact of their monitoring efforts, and encourage patients to adherence to the practice(30).

Despite the lower levels of BP device ownership, participants who owned personal BP devices were significantly more likely to monitor their BP within the community. These findings align with results from other studies conducted in both low- and middle-income countries (LMICs) and high-income countries (HICs)(31, 32). Owning a BP device addresses common barriers related to accessibility and convenience, facilitating regular community BP monitoring(32). Participants taking three or more medications were more than two folds likely to monitor their BP in the community compared to those who were receiving one anti-hypertensive medications. These findings is consistent with results from a study conducted in the United Kingdom where an increase in the number of anti-hypertensive medication was associated with a ten percent increase in likelihood to monitor BP (33). In stepwise management of hypertension, patients are prescribed three or more classes of medications if they have failed to achieve BP control on one or two classes of medications and often have hard to treat hypertension. These patients are often given additional attention, thoroughly educated about their condition, and encouraged to monitor their BP regularly. Moreover, such patients may be more motivated to monitor their BP, anticipating improvements in their readings.

One strength of this study is that the study examined the prevalence of BP monitoring using various approaches beyond self-BP monitoring. In addition, the study recruited participants from diverse settings representing both the peri and urban communities.

One of the limitations of this paper is that, it conducted at urban and peri-urban clinics and these results might not be generalizable to the rural settings, which are often less equipped to provide hypertension services(34). Secondly, hypertension care was integrated with HIV treatment in the study clinics, with most participants diagnosed with hypertension within the past year, underrepresenting individuals on long-term hypertension treatment. This study setting does not fully reflect real-world conditions, limiting generalizability. However, evidence suggests that participants who adopt similar practices are highly likely to sustain them over the long term(35). This implies that similar or nearly similar rates of community BP monitoring could be observed in more experienced individuals with hypertension. Thirdly, while there was potential for recall bias, efforts were made to minimize it by requesting documents to verify the information. The study’s quantitative approach also limits insight into contextual factors influencing community BP monitoring, which a mixed-methods approach could have addressed.

## CONCLUSIONS

Community BPM uptake among PLHIV and hypertension was low. However, individuals receiving healthcare provider recommendations and feedback, owning BP devices, or on three antihypertensive medications were more likely to engage in community BPM. To improve uptake of BP monitoring and ensure continuity of care, targeted interventions such as healthcare provider counseling, feedback mechanisms, and facilitating access to BP devices are recommended. In addition, private community medical facilities and pharmacies could be leveraged to support blood pressure monitoring and ensure continuity of hypertension care.

## Supplementary Material

This is a list of supplementary files associated with this preprint. Click to download.

• Supplementmaterial1.doc

## Figures and Tables

**Table 1:** Distribution of sample size across the three study sites.

Clinic	Location	Government level clinic	No of PLHIV with hypertension during Jan- March 2024	Proportion of PLHIV with hypertensio n at each clinic	Estimated sample size per clinic	Number enrolled
Kawaala	Urban	HCIV	651	69.3	282	282
Butabika	Urban	Referral hospital	156	16.6	67	67
Nakawuka	Peri-urban	HCIII	132	14.1	57	59
**Total**			**939**	**100**	**406**	**408**

**Table 2:** Socio-demographic characteristics of participants

	N (%) or Mean (SD) or Median (IQR)			
	Overall	Kawaala	Butabika	Nakawuka
	(n=408)	(n=282)	(n=67)	(n=59)
**Age [mean (SD), n=406, years]**	50.1 (10.7)	50.0 (10.2)	52.3 (10.3)	48.1 (13.2) *
18-35	37 (9.1)	21 (7.5)	4 (6.0)	12 (20.3) *
36-50	173 (42.4)	127 (45.0)	22 (32.8)	24 (40.7)
≥ 51	196 (48.0)	133 (47.2)	40 (59.7)	23 (39)
Missing	2 (0.5)	1 (0.4)	1 (1.5)	0 (0.0)
**Sex**				
Male	132 (32.3)	95 (33.7)	24 (35.8)	13 (22.0)
Female	276 (67.7)	187 (66.3)	43 (64.2)	46 (78)
**Distance to primary clinic (n=276), KM**	4 (3.0 −7.0)	5 (3.0-8.0)	1.3 (1.0-	3.0 (1.5-6.0)
**Distance to a nearby medical facility**	0.8 (0.3-1)	1.0 (0.3-	1 (0.3-1)	0.3 (0.2-0.8)
**Education level**				
Never attended school	77 (18.9)	67 (23.8)	2 (3.0)	8 (14.6) ***
Primary school	138 (33.8)	79 (28.0)	23 (34.3)	36 (61.0)
Secondary school	134 (32.8)	89 (31.6)	32 (47.8)	13 (22.0)
Post-secondary education	59 (14.5)	47 (16.7)	10 (14.9)	2 (3.4)
**Marital status**				
Married/Cohabiting	189 (46.3)	132 (46.8)	38 (56.7)	19 (32.2) ***
Single (Never married)	58 (14.2)	57 (20.2)	1 (1.5)	0 (0.0)
Divorced/ separated	92 (22.6)	53 (18.8)	18 (26.9)	21 (35.6)
Widowed	69 (16.9)	40 (14.2)	10 (14.9)	19 (32.2)
**Occupation**				
Formally employed	68 (16.7)	57 (20.2)	9 (13.4)	2 (3.4) ***
Self-employed	188 (46.1)	147 (52.1)	27 (40.3)	14 (23.7)
Peasant farmer	34 (8.3)	15 (5.3)	6 (9.0)	13 (22)
Unemployed	97 (23.8)	62 (22.0)	20 (29.9)	15 (25.4)
Others specify	21 (5.1)	1 (0.4)	5 (7.5)	15 (25.4)
**Monthly income**† **[(n=219), `000)**	300(122-	550 (300-	200 (40-	100 (20-280)
0-50000	40 (18.3)	2 (1.8)	14 (26.9)	24 (44.4) ***
50001-250000	56 (25.6)	23 (20.4)	17 (32.7)	16 (29.6)
250001- 500000	48 (21.9)	28 (24.8)	10 (19.2)	10 (18.5)
500001+	75 (34.3)	60 (21.2)	11 (21.2)	4 (7.4)
**Current smoker**	23 (5.6)	20 (7.1)	2 (3.0)	1 (1.7)
**Past smoker (n=385)**	55 (14.3)	42 (16.0)	6 (9.2)	7 (12.1)
**Alcohol consumption**				
Never	190 (46.6)	132 (46.8)	36 (53.7)	22 (37.3) **
Monthly or less	83 (20.3)	67 (23.8)	4 (6.0)	12 (20.3)
2-4 times a month	35 (8.6)	28 (9.9)	3 (4.5)	4 (6.8)
2-3 times a week	23 (5.6)	8 (2.8)	7 (10.5)	8 (13.5)
4 or more times a week	9 (21.2)	4 (1.4)	3 (4.5)	2 (3.4)
Used to drink but stopped	68 (16.7)	43 (15.3)	14 (20.9)	11 (18.6)

† Currency reported in Uganda shilling,

* p<0.05

** p<0.01

*** p<0.001

**Table 3:** Clinical characteristics of participants

Variable	N (%) or Mean (SD) or
**Current ART regimen**	
TDF/3TC/DTG	376 (92.2)
TDF/3TC/EFV	15 (3.7)
Others*	17 (4.1)
**Time spent on ART [Median (IQR), years]**	5 (3.5-7.2)
< 5 years	209 (51.2)
≥ 5 years	192 (47.1)
Missing	7(1.7)
**HIV DSD model** **	
FTDR	166 (40.7)
FBIM	65 (15.9)
CCLAD	4 (1.0)
CDDP	27 (6.6)
FBG	144 (35.3)
Missing	2 (0.5)
**Viral load status**	
Viral load suppressed	354 (86.8)
Viral load detectable (200-< 1000)	22 (5.4)
Viral load detectable (≥ 1000)	15 (3.7)
Unknown/Not done	17 (4.2)
**Had family history of hypertension**	**246 (60.3)**
Father (n=246)	64 (26)
Mother	94 (38.2)
Sibling	94 (52.8)
**Duration of hypertension diagnosis**	1 (1.0-1.0)
Less than 1 year	64 (15.7)
1 year	231 (56.6)
2 or more years	113 (27.7)
**Number of hypertension medications**	
One	184 (45.1)
Two	217 (53.2)
Three	7 (1.7)
**Time spent on hypertension medicines**	**1 (0.0 – 1.0)**
Less than 2 years	322 (78.9)
2 or more years	86 (21.1)
**Body mass index, kg/m^2^**	
Normal (18.5-<25)	148 (36.3)
Underweight (<18.5)	20 (4.9)
Overweight (25- <30)	125 (30.6)
Obese (≥ 30)	108 (26.5)
Missing	7 (1.7)
**BP status on previous clinic visit**	
Controlled BP***	**137 (33.6)**
Uncontrolled BP	**271 (66.4)**
**Presence of comorbid conditions**	**41 (10.1)**
Diabetes	26 (6.4)
Heart condition	2 (0.5)
Stroke	8 (2.0)
**Time since the last clinic visit [Median**	3 (2.4 - 4.0)

* AZT/3TC/ATV/r (2), TDF/3TC/ATV/r (1), ABC/3TC/DTG (10), ABC/3TC/EFV (2), AZT/3TC/DTG (2)

** FTDR; Fast Track Drug refill, FBIM; Facility-based individual management, Facility-based individual management, FBG; Facility-based groups, CCLAD;

**Table 4:** Behavioral and healthcare system-related factors

Variable	N (%) or Mean (SD) or
**Told by a healthcare provider to monitor BP in the**	
Yes	305 (74.7)
No	101 (24.8)
Missing	2 (0.5)
**Thought it is important to regularly monitor BP from**	
Yes	356 (87.3)
No	21 (5.2)
Missing	31 (7.6)
**Had a card or book where BP measurement can be**	
Yes	254 (62.3)
No	144 (35.3)
Missing	10 (2.4)
**Asked by the health care provider about community BP**	
Yes	222 (54.4)
No	178 (43.6)
Missing	8 (2.0)
**Received feedback on community BP monitoring**	
Yes	186 (45.6)
No	191 (46.8)
Missing	31 (7.6)
**Had treatment supporter**	
Yes	268 (65.7)
No	136 (33.3)
Missing	4 (1.0)
**Reasons for not monitoring BP while away from the**	
It is expensive.	139 (47.0)
Do not have time to measure BP	92 (31.1)
Do not have a personal BP device	41 (13.9)
Feared people to know about their HIV status	19 (6.4)
Feared people to know about their hypertension status	8 (2.7)
Never been told	71 (24.0)
**BP monitoring device ownership**	
Yes	16 (3.9)
No	391 (96.1)
**Reasons for not owning a personal BP monitor device**	
Too expensive / Unable to afford it	289 (73.9)
Not able to find one	47 (12.0)
Do not think I need one /Never thought about having	49 (12.5)
Not sure how to use it /too complicated	35 (9.0)
I have never been told about owning one	103 (26.3)
**Attitude score, median (IQR)**	22 (19-24)
**Knowledge about BP treatment goal**	
Knows the BP treatment goal	92 (22.5)
Does not know the treatment goal	316 (77.5)
**Knowledge about hypertension complication**	
Knows at least one complication	261 (64.0)
Does not know any complication	147 (36.0)
**Knowledge about BP monitoring**	
0-2	137 (33.6)
3-4	271 (66.4)

**Table 5:** Prevalence of community BP monitoring

Variable	N (%) or Mean (SD) or Median (IQR)	95% CI
**Monitored/measured BP in the community**		
Yes	112 (27.5)	23.3 – 32 %
No	296 (72.5)	68.0- 76.7 %
**Number of times community BP monitoring**	3(2.0 - 4.0)	N/A
**Frequency of community BP monitoring**		
Daily	7 (6.3)	3.0 −12.7 %
2-3 times a week	15 (13.4)	8.2.1-21.2 %
Once a week	9 (8.0)	4.2-14.9 %
4-6 times a week	6 (5.5)	2.5 −42.4 %
Once a month	37 (33.0)	24.9 – 42.4%
1-3 times a month	33 (29.5)	21.7 −38.7 %
Never	3 (2.7)	0.9 – 8.1%
Others	2 (1.8)	0.5 – 7.0%
**Places where participants monitored their**		
Private clinic	77 (68.6)	59.5 – 76.7 %
Pharmacy	79(8.0)	4.2 – 14.9 %
Other public health facility other the	17 (15.2)	9.6 – 23.2 %
Home, using a self-owned BP device	6 (5.4)	2.4 – 11.5%
From home, using a friend's BP devices	2 (1.8)	0.4- 7.0%
**Community BP**		
**Mean SBP n=98**	137.2 (20.2)	NA
**Mean DBP n=96**	85.8 (13.2)	NA
**Paid to monitor BP (n=111)**	70 (63.1)	
Amount paid*(n=111)	2,000 (1,000-2,000)	

* Currency reported in Uganda shilling, NA-Not applicable, BP- Blood pressure, SBP -systolic blood pressure, DBP – Diastolic blood pressure

**Table 6:** Bivariable and multivariable analysis of factors associated with uptake of community BP monitoring

Variable	Yes, n (%)	No, n (%)	Unadjusted PR (95% CI)	Adjusted PR (95% CI)
**Age**				
18-35	9 (24.3)	28 (75.7)	Ref	Ref
36-50	48 (27.8)	125 (72.2)	1.1 (0.6-2.3)	1.0 (0.8-1.2)
≥ 51	55 (28.1)	141 (71.9)	1.2 (0.6-2.3)	0.8 (0.4-1.4)
**Sex**				
Male	85 (30.8)	191 (69.2)	Ref	Ref
Female	27 (20.5)	105 (79.5)	0.7 (0.4-1.0)	0.8 (0.6-1.1)
**Monthly income (n=91, `000)**				
0-50000	11 (27.5)	29 (72.5)	Ref	Ref
50001-250000	14 (25.0)	42 (75.0)	0.9 (0.4-2.0)	0.9(0.6-1.3)
250001- 500000	6 (12.5)	42 (87.5)	0.5 (0.2-1.2)	0.8 (0.7-1.1)
500001+	20 (26.7)	55 (73.3)	1.0 (0.5-2.0)	1.2 (0.6-2.5)
**Presence of comorbid conditions**				
No	97 (26.4)	270 (73.6)	Ref	Ref
Yes	15 (36.6)	26 (63.4)	1.4 (0.8-2.4)	1.2 (0.9-2.5)
**BP status on previous clinic visit**				
Uncontrolled BP†	76 (28.0)	195 (72.0)	Ref	Ref
Controlled BP	36 (26.3)	101 (73.7)	0.9 (0.6-1.4)	0.8(0.4-1.5)
**Number of hypertension**				
1	51 (27.7)	133 (72.3)	Ref	Ref
2	57 (26.3)	160 (73.7)	0.9 (0.6-1.4)	1.5 (0.9-2.5)
3	4 (57.1)	3 (42.9)	2.1 (0.7-5.7)	2.2 (1.1-4.4) *
**Knowledge about hypertension**				
Doesn’t know BP goals	78 (24.7)	238 (75.3)	Ref	Ref
Knows BP goals	34 (37.0)	58 (63.0)	1.5 (1.0-2.2) *	1.1 (0.5-2.4)
**Knowledge about hypertension**				
Does not know any complication	31 (21.1)	116 (78.9)	Ref	Ref
Knows at least one complication	81 (31.0)	180 (69.0)	1.5 (1.0-2.2)	0.7 (0.5-1.0)
**Knowledge about BP monitoring**				
0-2	16 (11.7)	121 (88.3)	Ref	Ref
3-4	96 (35.4)	175 (64.6)	3.0 (1.8-5.1) ***	1.5 (0.6-4.2)
**Attitude score**				
Poor	2 (4.4)	43 (95.6)	Ref	Ref
Good	106 (30.2)	245 (69.8)	6.8(1.6-27.5) **	0.2 (0.0-2.7)
**Has a card or book for CBP**				
No	9 (6.2)	135 (93.8)	Ref	Ref
Yes	103 (40.5)	151 (59.5)	6.5 (3.2-12.8) ***	1.4 (0.6-3.2)
**Received advice about CBP from**				
No	10 (9.9)	91 (90.1)	Ref	Ref
Yes	102 (33.4)	203 (66.6)	3.4 (1.8-6.4) ***	8.7 (3.7-20.5)
**BP ownership**				
No	101 (25.8)	290 (74.2)	Ref	Ref
Yes	11 (68.8)	5 (31.3)	2.6 (1.4-5.0) ***	2.2 (1.3-3.5)
**Duration on ART**				
0-<5	47 (22.5)	162 (77.5)	Ref	Ref
≥5 year	61 (31.8)	131 (68.2)	1. 4(1.0-2.1)	1.1 (0.5-2.8)
**Provider reviews patient’s**				
No	6 (3.4)	172 (96.6)	Ref	Ref
Yes	105 (47.3)	117 (52.7)	14.0(6.2-31.9) ***	0.8(0.5-1.1)
**Provider provides feedback about**				
No	3 (1.6)	188 (98.4)	Ref	Ref
Yes	107 (57.5)	79 (42.5)	36.6 (11.6-115.4)	33.3(4.1-

* p<0.05

** p<0.01

*** p<0.001

† BP control defined as systolic BP < 140 and Diastolic < 90

Variables adjusted for include clinic name, age category, monthly income, BP status

## Abbreviations:

AIDS: Acquired immunodeficiency syndrome
ART: antiretroviral therapy
BMI: Body mass index
BP: Blood pressure
BPM: Blood pressure monitoring
CVD: Cardiovascular diseases
HBPM: Home Blood pressure monitoring
HC: Health center
HCP: Healthcare providers
HIC: High income countries
HIV: Human Immunodeficiency Syndrome
HTN: Hypertension
IQR: Inter Quartile Range
LMICs: Lower-middle-income countries
MMD: Multi-month drug
MoH: Ministry of Health
NCD: Non-communicable disease
PHC: Primary Healthcare
PLHIV: People living with HIV
PNFP: Private not for profit
SD: Standard deviation
STROBE: Strengthening the Reporting of Observational Studies in Epidemiology
REDCap: Research Electronic Data Capture
WHO: World Health Organization
